# Case report: ^18^F-FES PET/CT predicted treatment responses of second-line and third-line CDK4/6 inhibitors after disease progression on first-line CDK4/6 inhibitor in a HR+/HER2- metastatic breast cancer patient

**DOI:** 10.3389/fonc.2022.1095779

**Published:** 2022-12-23

**Authors:** Bo Pan, Zhixin Hao, Ying Xu, Zhe Wang, Ru Yao, Xuefei Wang, Chao Ren, Yidong Zhou, Qiang Sun, Li Huo

**Affiliations:** ^1^ Department of Breast Surgery, Peking Union Medical College Hospital, Chinese Academy of Medical Sciences and Peking Union Medical College, Beijing, China; ^2^ Department of Nuclear Medicine, Peking Union Medical College Hospital, Chinese Academy of Medical Sciences and Peking Union Medical College, Beijing, China; ^3^ Beijing Key Laboratory of Molecular Targeted Diagnosis and Therapy in Nuclear Medicine, Beijing, China

**Keywords:** metastatic breast cancer, FES PET/CT, estrogen receptor (ER), CDK4/6 inhibitor, treatment response

## Abstract

**Background:**

Cyclin-dependent kinase 4/6 inhibitor (CDK4/6i) has become the commonest first-line treatment of hormonal receptor positive and human epidermal growth factor receptor 2 negative (HR+/HER2-) metastatic breast cancer (MBC). However, therapy is quite individualized after progression of disease (PD) when CDK4/6i fails. Estrogen receptor (ER) status of metastatic lesions of bone, lung or liver might be different from the primary tumor and biopsy of metastatic lesions was invasive and not always available. Prediction of treatment response after PD of CDK4/6i remains unsolved. ^18^F-fluoroestradiol (FES) PET/CT could non-invasively reveal ER expression both in primary and metastatic breast cancer and recognize heterogeneity of ER status.

**Case presentation:**

A 70-year-old woman with Parkinson’s disease, osteoporosis and cardiovascular co-morbidity was diagnosed with HR+/HER2- breast cancer (pT2N2M0, stage IIIa). Three years later, she developed metastases in right lung and pleura with pleural effusion and received palbociclib + letrozole. After 8 months the disease progressed, and ^18^F-FES PET/CT revealed multiple ER-positive pleural lesions and ER-negative pulmonary nodules after PD and the progression-free survival (PFS) of first-line CDK4/6i was 8 months. Since most of the metastatic lesions were ER-positive, abemaciclib + fulvestrant were chosen as the second-line CDK4/6i treatment and the PFS was 15 months. Another ^18^F-FES PET/CT showed a new ER-positive pleural mass with multiple ER-negative pulmonary nodules. Since ^18^F-FES PET/CT revealed that the dominant lesions were still ER-positive, dalpiciclib + exemestane + fulvestrant were prescribed as the third-line CDK4/6i treatment. Currently the patient’s disease had been stable for 2 months.

**Conclusion:**

This case demonstrated that ^18^F-FES PET/CT could show ER heterogeneity non-invasively and reveal the treatment responses a predictive imaging tool of serial second- and third-line of CDK4/6i treatments when first-line CDK4/6i failed in HR+/HER2- MBC. So long as the dominant or newly-developed metastatic lesion was ER-positive on ^18^F-FES PET after first-line CDK4/6i, the patient might show certain therapeutic response towards endocrine-based treatment including second- and third-line of CDK4/6i, and thus increased the time to chemotherapy (TTC).

## 1 Introduction

Breast cancer (BC) is the commonest malignancy worldwide and the leading cause of cancer death in Chinese women younger than 45 years (1–3). Cyclin-dependent kinase 4/6 inhibitor (CDK4/6i) has become the standard first-line treatment of hormonal receptor positive and human epidermal growth factor receptor 2 negative (HR+/HER2-) metastatic breast cancer (MBC) (4, 5). However, the treatment for the progression of disease (PD) after CDK4/6i fails would be quite diversified and individualized (6). ^18^F-fluorodeoxyglucose (^18^F-FDG) is the most commonly used PET tracer for metastatic examination, management response, and suspected recurrence of BC (7). However, based on increased glycolysis and glucose transporters of tumor cells, ^18^F-FDG is not a cancer-specific tracer. Estrogen receptor (ER) is over-expressed in approximately 70% of breast malignancies and an important target for endocrine therapy (8). ^18^F-fluoroestradiol (^18^F-FES) is a radiotracer binding to ER, and allows non-invasive, whole-body evaluation of ER expression in BC (9).With its potential to serve as a prognostic and predictive biomarker for hormonal therapy of BC, ^18^F-FES PET has gained growing interest in research (10). Here we report a case of HR+/HER2- MBC patient whose serial ^18^F-FES PET/CT revealed treatment responses of second-line and third-line CDK4/6i + endocrine therapy after PD on first-line CDK4/6i + endocrine therapy. ^18^F-FES PET/CT may act as a non-invasively predictive imaging tool to guide subsequent treatments when first-line CDK4/6i failed in MBC patients.

## 2 Case presentation

### 2.1 Surgery and postoperative adjuvant therapy of the primary tumor

A 70-year-old woman noticed a palpable mass in her left breast for three months and came to the hospital in December 2016. Her past medical history included Parkinson’s disease, osteoporosis, and cardiovascular co-morbidities such as mild ischemic heart disease and lacunar infarctions of brain, and she took medications accordingly. She has no family history of BC. On physical examination, she could walk and talk slowly with continuous tremor in left arm and hand and decreased facial expression which was typical as ‘masked face’ of Parkinson’s disease. There was a 2.5cm round hard lump in the left upper outer quadrant of the left breast without nipple discharge and palpated movable lymph nodes (LN) about 1.5cm in size in the left axilla. Ultrasound revealed a left breast solitary mass measuring 3.1×2.7cm^2^ and abnormal enlarged LN in the left axilla, infra- and supra-clavicular fossa, considering LN metastasis. The mammogram did not find clusters of micro-calcifications. Multi-disciplinary consultation indicated that the risk of general anesthesia for the patient was relatively high. Both the patient and her relatives preferred procedures under local anesthesia to ensure safety. Considering the abnormally enlarged supra-clavicular LN, the surgical procedure might be palliative instead of radical.

The patient underwent left breast extended lumpectomy under local anesthesia (LA) in January 2017. Intraoperative fast frozen biopsy revealed left BC so patient subsequently received sentinel and targeted axillary lymph node biopsy under LA on the same day. Post-operative pathology showed invasive breast carcinoma, not otherwise specified (NOS), measuring 2.3×1.5×1.5cm^3^, and LN metastasis (6/6). Immunohistochemistry (IHC) was ER (++, 95%), PR (++, 40%), HER2 (2+), fluorescence *in situ* hybridization (FISH) (-), and Ki-67 index 30%. The stage was pT2N2M0, IIIa, and molecular subtype was luminal B. With awareness of the risk of insufficient treatment and lack of evidence, the patient and her relatives refused intravenous chemotherapy and chose oral chemotherapy. Therefore she was put on adjuvant capecitabine (1.5g BID, 2 weeks on/1 week off) for 8 cycles. Concurrently, the patient underwent whole breast radiotherapy (WBRT) plus tumor bed boost, with the left axilla included. After the oral chemotherapy was finished, she received tamoxifen as endocrine therapy instead of aromatase inhibitor (AI) due to osteoporosis.

### 2.2 First-line CDK4/6i (palbociclib) treatment for metastasis

**Figure 1:**
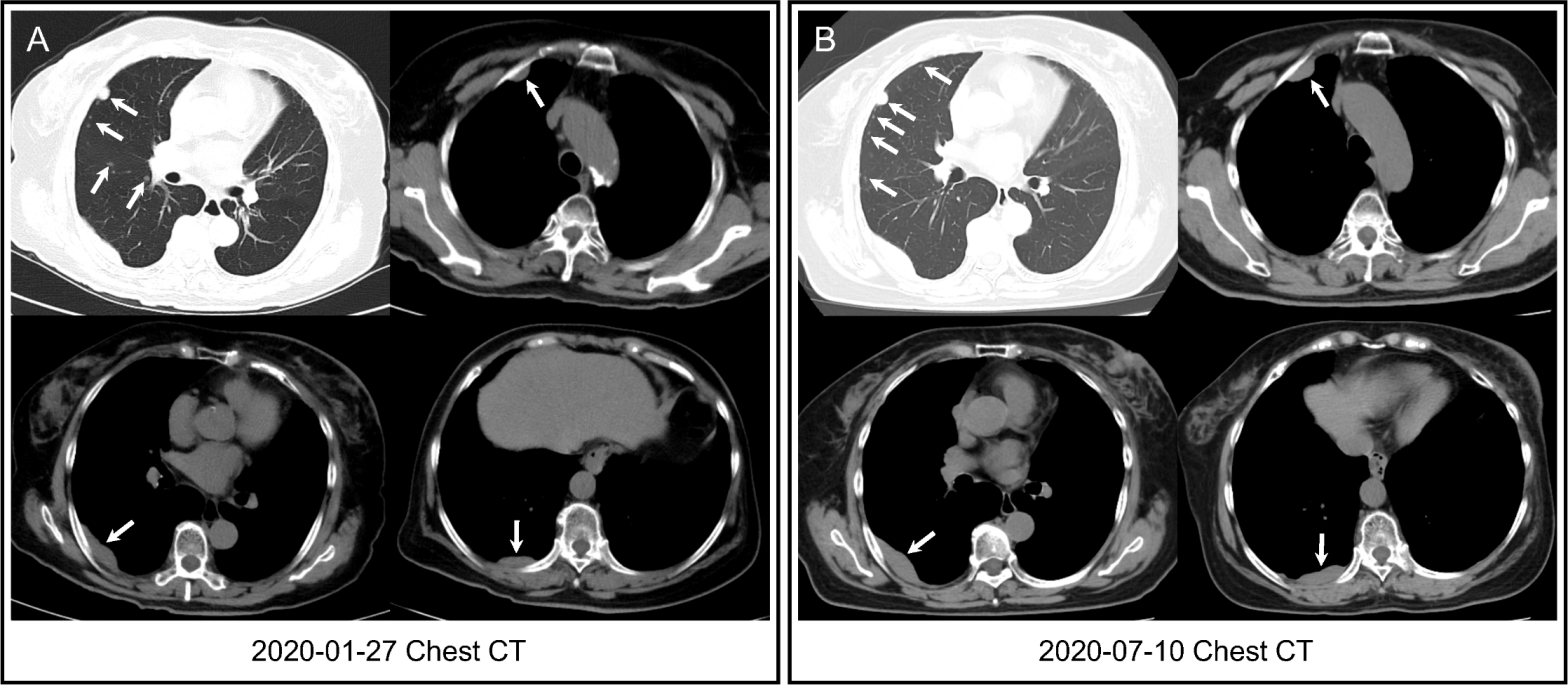
Chest CT. **(A)** Chest CT showed multiple pulmonary nodules in right lung and masses in right pleura (arrows) three years after surgery. **(B)** Followed-up chest CT revealed that pulmonary nodules increased in number, and pleural masses increased in size (arrows) when first-line CDK4/6i + endocrine therapy was started.

**Figure 2:**
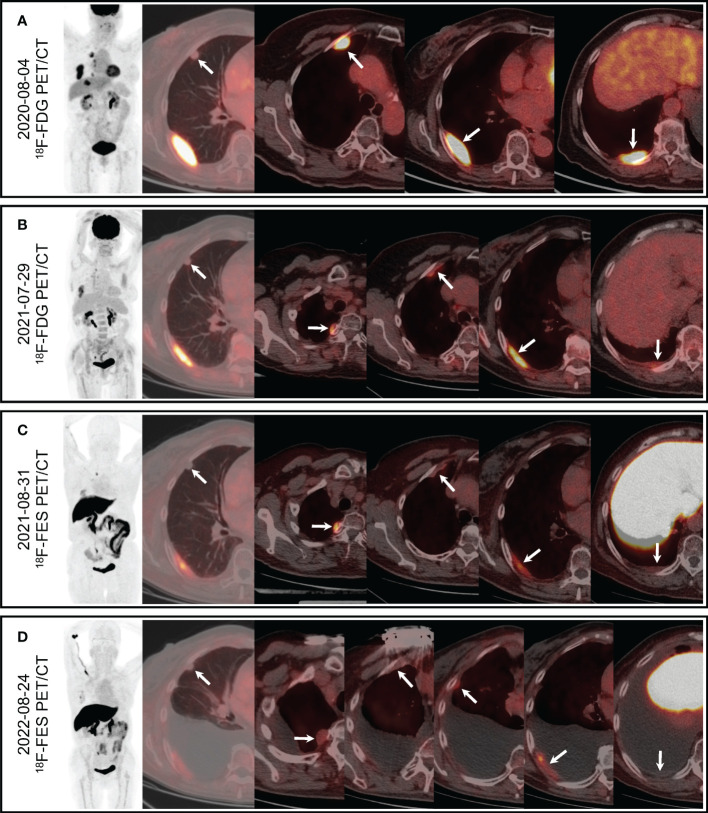
PET/CT. **(A)** ^18^F-FDG PET/CT showed mild FDG uptake of the largest pulmonary nodule (arrow, SUVmax 1.6) and increased FDG uptake of the multiple pleural masses (arrows, SUVmax 9.4). **(B)** Followed-up ^18^F-FDG PET/CT showed mild FDG uptake of the largest pulmonary nodule (arrow, SUVmax 1.2) and increased FDG uptake of the multiple pleural masses (arrows, SUVmax7.0). **(C)** Pulmonary nodule was negative on ^18^F-FES PET/CT (arrow), and multiple pleural masses showed obvious FES uptake (arrows, SUVmax 5.0). **(D)** Pulmonary nodule was negative on followed-up ^18^F-FESPET/CT (arrow), and multiple pleural masses showed different levels of FES uptake (arrows, SUVmax3.8).

The patient was followed every half-year post-operatively and the results were normal. However, in January 2020, her chest CT showed multiple pulmonary nodules in right lung (the largest measuring 12.2 mm) andmasses in right pleura (the largest measuring 5.0 × 1.4 cm^2^) (**Figure 1A**). She developed mild cough and dyspnea in July 2020, and followed-up chest CT showed the progression of the pulmonary nodules and masses in the right pleura (**Figure 1B**), suggesting pulmonary and pleural metastases. ^18^F-FDG PET/CT showed multiple FDG avid pleural masses with maximum standardized uptake value (SUVmax) 9.4, and multiple pulmonary nodules with mild FDG uptake (SUVmax1.6), demonstrating pulmonary and pleural metastases (**Figure 2A**). The metabolic tumor volume (MTV) of the largest pleural mass was 27.7 cm^3^. There were no metastases in bone, liver, or brain on ^18^F-FDG PET/CT. The imaging evaluation of the patient was summarized in **Table 1**. In August 2020, the patient received first-line CDK4/6i palbociclib (125mg QD, 3 weeks on/1 week off) + letrozole (2.5mgQD) and intravenous bisphosphonate (Q6M) with 1, 25-dihydroxyvitamin D3 to intensify the treatment of osteoporosis.

**Table 1:** Imaging evaluation of the patient.

Variables	Chest CT2020-01-27	Chest CT2020-07-10	^18^F-FDG PET/CT2020-08-04	Chest CT2020-12-24	Chest CT2021-04-27	^18^F-FDG PET/CT2021-07-29	^18^F-FES PET/CT2021-08-31	^18^F-FES PET/CT2022-08-24
Diameter of the maximum pulmonary nodule (mm)	12.2	11.0	11.3	9.4	10.1	9.6	9.5	9.7
SUVmax of the maximum pulmonary nodule	NA	NA	1.6	NA	NA	1.2	0.8	1.2
Size of the targeted pleural lesion (cm^2^)	5.0 × 1.4	5.3 × 1.3	5.2 × 1.5	4.3 × 1.3	4.4 × 1.2	4.0 × 1.2	3.8 × 1.0	4.8 × 1.1
SUVmaxof the targeted pleural lesion	NA	NA	9.4	NA	NA	7.0	3.6	3.8
MTV of the targeted pleural lesion (cm^3^)	NA	NA	27.7	NA	NA	13.9	NA	NA
EEV of the targeted pleural lesion (cm^3^)	NA	NA	NA	NA	NA	NA	17.2	5.3

CT, computed tomography; FDG, fluorodeoxyglucose; PET/CT, positron emission tomography/computed tomography; FES, fluoroestradiol; SUVmax, maximum standardized uptake value; MTV, metabolic tumor volume; EEV, estrogen receptor expression volume; NA, not available.

She suffered from mild delirium, delusion, progressive tremor of left upper extremity and grade 4 neutropenia after the first cycle of palbociclib, so the dose of palbociclib was reduced to 100mg QD (3 weeks on/1 week off) from the second cycle. All the above-mentioned adverse effects recovered partially yet remained. In December 2020, her discomfort had relieved, and the chest CT showed both the pulmonary nodules and pleural masses were smaller (the largest pulmonary nodule measuring 9.4 mm, and the largest pleural mass measuring 4.3 × 1.3 cm^2^), achieving partial remission (PR). However, her cough and dyspnea got worse in April 2021, and her chest CT revealed that pulmonary nodules and masses in pleura were larger than before (the largest pulmonary nodule measuring 10.1 mm, and the largest pleural mass measuring 4.4 × 1.2 cm^2^), and there was increasing pleural effusion, suggesting PD. The progression free survival (PFS) of first-line CDK4/6i was 8 months.

### 2.3 Second-line CDK4/6i (abemaciclib) treatment for metastasis and ^18^F-FES PET/CT

In May 2021, the patient started second-line CDK4/6i abemaciclib (150mg, BID) + fulvestrant (500mg, im, Q2W×3, then Q4W) after palbociclib + letrozole had failed. Two months later, ^18^F-FDG PET/CT was performed to evaluate the treatment response (**Figure 2B**), which revealed that both the pulmonary nodules and the pleural masses were smaller than that of chest CT in April 2021 (the largest pulmonary nodule measuring 9.6 mm, and the largest pleural mass measuring 4.0 × 1.2 cm^2^), and pleural effusion was decreased. SUVmax of the lesions as well as MTV of the largest pleural mass (13.9 cm^3^) were decreased than those of previous FDG PET/CT (**Table 1**)

To evaluate the ER status of metastatic lesions, ^18^F-FES PET/CT was performed subsequently (**Figure 2C**). The study was approved by the institutional ethics review board of the Peking Union Medical College (PUMC) Hospital (IRB protocol #JS-2959), and written informed consent was obtained. The ^18^F-FES PET/CT showed multiple ER-positive pleural masses with obvious FES uptake and multiple ER-negative pulmonary nodules, which implied ER heterogeneity of metastases. The ER expression volume (EEV) of the largest pleural mass was 17.2 cm^3^. The condition of the disease was partial remission (PR). Hence the treatment (abemaciclib + fulvestrant) was continued, and the tolerance was acceptable. She suffered adverse effects including grade 2 neutropenia and grade 1/2 diarrhea, without delirium or delusion, and the tremor was mitigated. Medications including bisphosphonate (iv, Q6M), 1, 25-dihydroxyvitamin D3 were also continued.

In August 2022, she felt uncomfortable and the dyspnea recurred. The second ^18^F-FES PET/CT was performed and there was a new ER-positive pleural mass with SUVmax 2.7 and EEV 5.3 cm^3^ (**Figure 2D**), and the other pleural metastases decreased both in size and uptake of FES. Multiple pulmonary nodules were still ER-negative, and the pleural effusion increased notably. The imaging evaluation suggested PD, and PFS of second-line CDK4/6i was 15 months.

### 2.4 Third-line CDK4/6i (dalpiciclib) treatment for metastasis

**Figure 3:**
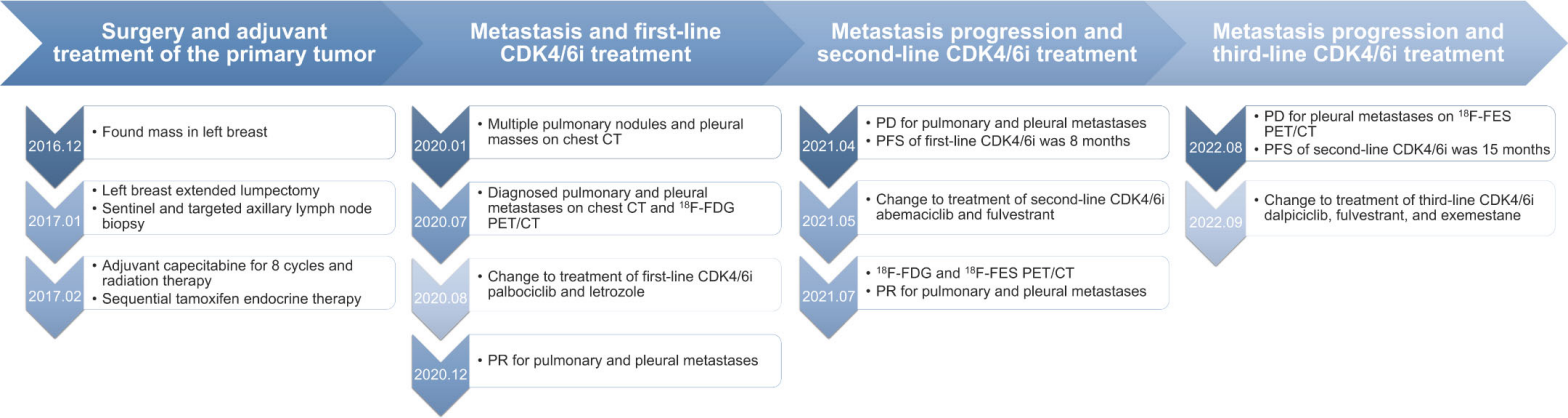
Timeline of the patient’s disease progression.

Since ^18^F-FES PET/CT revealed that the most of the metastatic lesions were still positive, the patient and her relatives turned down the option of intravenous or oral chemotherapy and were willing to try another line of endocrine-based therapy. In September 2022, the patient was put on dalpiciclib (125mg, QD, 3 weeks on/1 week off) + fulvestrant (500mg, im, Q4W) + exemestane (25mg, QD), and underwent the drainage of pleural effusion. She felt improved appetite, relieved fatigue afterwards, and laboratory test revealed grade 2/3 neutropenia. Currently her disease had been stable for 2 months after the third-line CDK4/6i. The timeline of the patient’s disease progression was summarized in **Figure 3**.

## 3 Discussion

Breast cancer (BC) is one the leading cause of cancer-related death in women worldwide and the five-year cancer-specific survival of MBC is no more than 40% (2, 3, 5, 11–13). HR+/HER2- subtype comprises approximately 60% of all BC, and hyperactivity of CDK4/6 pathway is a common characteristic of HR+/HER2- BC, leading to resistance to endocrine therapy (14, 15). CDK4/6i in combination with endocrine therapy as both first-line and second-line treatment have shown to improve survival outcomes in patients with HR+/HER2- MBC with accumulating evidence of clinical trials including the MONARCH (6, 16, 17), PALOMA (18, 19), MONALESSA (20–22) and DAWNA series (23, 24). Meta-analysis and pooled-analysis also confirmed the survival benefits of all CDK4/6i in combination with endocrine therapy for HR+/HER2- MBC patients (25, 26).

ER status of BC could be assessed by PET/CT with ^18^F-FES (9, 11, 27), which binds to ER *in vivo* and enables imaging of ER expression both in primary and metastatic tumors non-invasively (11, 28). Based on the recommendations of ^18^F-FES PET to image the ER *in vivo* and clinical studies that correlated 18F-FES PET with IHC, a lesion with SUVmax >1.5 should be considered ER positive (8). A prospective study has shown high agreement between ^18^F-FES PET/CT result and ER status by IHC (11). Another large prospective trial also demonstrated high diagnostic accuracy of ^18^F-FES PET/CT, with a sensitivity of 95%, a specificity of 80%, a positive predictive value of 93%, and a negative predictive value of 85% (28). Therefore, ^18^F-FES PET/CT is a valid non-invasive imaging modality to determine ER status in MBC patients.

Furthermore, multiple potential clinical applications for ^18^F-FES PET have been proposed, including selecting patients for hormonal therapy, solving clinical dilemmas and systemic staging of tumors with low metabolic activity (10). In terms of prediction of therapeutic response, study had shown that patients with ER heterogeneity or uncertainty tumors on ^18^F-FES PET showed better sensitivity to chemotherapy rather than endocrine therapy (29). In another trial evaluating tumor heterogeneity by ^18^F-FES PET as a predictive marker in MBC patients receiving palbociclib combined endocrine therapy, nine out of ten patients with an FES-negative site developed PD, with a median PFS of only 2.4 months. Among 46 patients with only FES-positive lesions, only four patients developed PD, with a median PFS of 23.6 months. Hence ^18^F-FES-PET may provide a promising method for identifying and selecting candidate ER+/HER2- MBC patients who would most likely benefit from CDK4/6i combined with endocrine treatment (30).

However, detection of hepatic metastases is known to be difficult on ^18^F-FES PET because of physiologic excretion of ^18^F-FES by the liver (8, 31). For this reason, patients need to be evaluated by contrast-enhanced CT, MRI, or ^18^F-FDG PET to detect hepatic lesions and ^18^F-FES PET is used to evaluate ER status of detected hepatic lesions. Homogeneous uptake suggests the absence of lesions, but ER-positive lesions with similar uptake as the liver cannot be excluded; “hot spots” indicate ER-positive lesions; and “cold spots” indicate benign cysts, ER-negative lesions, but also lesions with low ER expression or even high expression (8, 31).

Our patient suffered from metastases three years after surgery, partly due to the insufficient post-operative adjuvant therapy. In addition to chemotherapy, she might also potentially benefit from intensified adjuvant treatment of abemaciclib, which was not available until April 2021. In consideration of the ER-positive pleural masses and ER-negative pulmonary nodules, the metastases of the lady showed ER heterogeneity. Notably, she showed the acceptable therapeutic effect of second-line CDK4/6i + fulvestrant after the first-line CDK4/6i + aromatase inhibitor (AI). The mechanism leading to CDK4/6i resistance included the loss of drug target genes such as RB and FZR1, the over-expression of genes which are involved in the progression of cell cycle, the over-expression of factors which are upstream of the cell cycle such as FGFR, PI3K/AKT/mTOR, and the TGF-β induced expression of several transcription factors involved in epithelial-mesenchymal transition (EMT) *via* Smad and the PI3K/AKT/mTOR pathways (32). Accordingly, the treatment choice after CDK4/6i fails included endocrine therapy in combination with mTOR inhibitor (e.g. everolimus) (33, 34), PI3K inhibitor (e.g. alpelisib) (35), another CDK4/6i (e.g. ribociclib, as in MAITAIN trial) (36) and HDAC (e.g. chidamide) (34). Antibody-drug conjugates (ADC) and chemotherapy would also be reasonable options under certain circumstances. Particularly, our patient was very reluctant to receive chemotherapy. She refused intravenous chemotherapy directly. We chose CDK4/6i as second- and third-line treatment to save other treatment methods with certain PFS for later use and to increase the time to chemotherapy (TTC). Therefore, so long as the dominant or newly-developed metastatic lesion was ER-positive on ^18^F-FES PET, the patient would show certain therapeutic response towards endocrine-based treatment including second- and third-line of CDK4/6i.

## 4 Conclusion

This case demonstrated that ^18^F-FES PET/CT could non-invasively show ER heterogeneity and predict the treatment response of second- and third-line CDK4/6i treatment in HR+/HER2- MBC after the first-line CDK4/6i failed. So long as the dominant metastatic lesion was ER-positive on ^18^F-FES PET, the patient would show certain therapeutic response towards endocrine-based treatment and the time to chemotherapy (TTC) might be increased.

## Data availability statement

The raw data supporting the conclusions of this article will be made available by the authors, without undue reservation.

## Ethics statement

The studies involving human participants were reviewed and approved by Ethics review board of the Peking Union Medical College (PUMC) Hospital (IRB protocol #JS-2959). The patients/participants provided their written informed consent to participate in this study. Written informed consent was obtained from the individual(s) for the publication of any potentially identifiable images or data included in this article.

## Author contributions

BP, ZH, and YX designed the study and wrote the first draft of the manuscript. ZW, RY, and XW made contributions to the acquisition of the clinical data. YZ, QS, and LH made critical revisions and approved final version. All authors contributed to the article and approved the submitted version.
